# Posttraumatic Stress Disorder and Neuroprogression in Women Following Sexual Assault: Protocol for a Randomized Clinical Trial Evaluating Allostatic Load and Aging Process Acceleration

**DOI:** 10.2196/19162

**Published:** 2020-11-18

**Authors:** Bruno Messina Coimbra, Mary Yeh, Ana Teresa D'Elia, Mariana Rangel Maciel, Carolina Muniz Carvalho, Ana Carolina Milani, Adriana Mozzambani, Mario Juruena, Sintia Iole Belangero, Andrea Parolin Jackowski, Dalva Poyares, Andrea Feijo Mello, Marcelo Feijo Mello

**Affiliations:** 1 Department of Psychiatry Universidade Federal de São Paulo São Paulo Brazil; 2 Program for Research and Care on Violence and PTSD Universidade Federal de São Paulo São Paulo Brazil; 3 Department of Morphology and Genetics Universidade Federal de São Paulo São Paulo Brazil; 4 Laboratory of Integrative Neuroscience Universidade Federal de São Paulo São Paulo Brazil; 5 Centre for Affective Disorders, Department of Psychological Medicine, Institute of Psychiatry, Psychology and Neuroscience, Biomedical Research Centre, South London and Maudsley NHS Foundation Trust and King's College London United Kingdom; 6 Department of Psychobiology Universidade Federal de São Paulo São Paulo Brazil

**Keywords:** PTSD, neuroprogression, allostatic load, sexual assault, trauma, thematic research, randomized clinical trial, aging

## Abstract

**Background:**

Posttraumatic stress disorder (PTSD) is a prevalent, chronic, and severe disorder related to traumatic events. Women are disproportionately affected by PTSD than men and are more at risk in the occurrence of sexual assault victimization. Estimates suggest that 50% of women develop PTSD following sexual assault and successful clinical management can be challenging. Growing evidence has implicated neural, immune, and endocrine alterations underpinning PTSD, but only few studies have assessed the evolution of acute PTSD in women.

**Objective:**

This study aims to measure whether the onset of PTSD is associated with accelerated aging in women following sexual assault. We hypothesize that the increase of allostatic load caused by PTSD leads to neuroprogression. We will implement a randomized clinical trial to compare responses to treatment with either interpersonal psychotherapy adapted for PTSD (IPT-PTSD) or the selective serotonin reuptake inhibitor sertraline.

**Methods:**

We will include women between 18 and 45 years of age, who experienced sexual assault from 1 to 6 months before the initial evaluation, and present with a Diagnostic and Statistical Manual of Mental Disorders, fifth edition (DSM-5) diagnosis of PTSD. Baseline evaluation will comprise clinical and psychometric assessments, structural and functional magnetic resonance imaging, neuropsychological testing, polysomnography, evaluation of immune and endocrine parameters, and genetic analyses. Age-matched female healthy controls will be included and subjected to the same evaluation. Patients will be randomized for treatment in 1 of the 2 arms of the study for 14 weeks; follow-up will continue until 1 year after inclusion via treatment as usual. The researchers will collect clinical and laboratory data during periodic clinical assessments up to 1-year follow-up.

**Results:**

Data collection started in early 2016 and will be completed by the end of the first semester of 2020. Analyses will be performed soon afterward, followed by the elaboration of several articles. Articles will be submitted in early 2021. This research project has obtained a grant from the Fundação de Amparo à Pesquisa do Estado de São Paulo (FAPESP 2014/12559-5).

**Conclusions:**

We expect to provide insight into the consequences of recent sexual assault exposure in women by investigating the degree of neuroprogression developing from an early stage of PTSD. We also expect to provide important evidence on the efficacy of a non-exposure psychotherapy (IPT-PTSD) to mitigate PTSD symptoms in recently sexually assaulted women. Further, we aim to obtain evidence on how treatment outcomes are associated with neuroprogression measures.

**Trial Registration:**

Brazilian Clinical Trials Registry RBR-3z474z; http://www.ensaiosclinicos.gov.br/rg/RBR-3z474z/

**International Registered Report Identifier (IRRID):**

DERR1-10.2196/19162

## Introduction

### Background

Posttraumatic stress disorder (PTSD) is a psychiatric disorder triggered by an external traumatic event that exposes a person to an imminent life-threatening situation [1]. PTSD is characterized by intrusive memories and thoughts about the traumatic event, avoidance of trauma-related reminders and feelings, negative alterations in cognition and mood, and hyperarousal [2]. Although trauma is a necessary condition for PTSD diagnosis, not all traumatized individuals will develop the disorder [3]. Those who develop PTSD may show elevated risk of health decline (eg, diabetes, cardiovascular disease, autoimmune diseases, hypertension, and dementia) and comorbidity with other psychiatric disorders [4,5]. PTSD is a significant societal burden, with increased likelihood of hospitalization, suicide, drug abuse, and aggressive behavior [6-8].

PTSD etiology is complex and is underpinned by the integration of endogenous and environmental factors. Molecular genetics–based heritability and epigenetic influences associated with adversities during childhood reflect this integration [9,10]. Women are more likely than men to develop PTSD following trauma in a female-to-male ratio of approximately 2:1 [11,12]. Women also have more prolonged and poorer PTSD outcomes than men regardless of threat or injury event–related factors [13]. The reasons for this gender discrepancy remain unclear, but evidence suggests that psychosocial, cultural, and biological factors play significant roles in the increased vulnerability of women to disorder onset and progression [14,15]. Among traumatic events that may challenge individuals in their lifetime, sexual trauma appears a major instigator of developing PTSD. Approximately half of women will develop PTSD following sexual assault [16].

### Allostatic Load and Neuroprogression in PTSD

PTSD has evolved its concepts in the Diagnostic and Statistical Manual of Mental Disorders, fifth edition (DSM-5) to include a complex heterogeneity of symptom profiles that may impede successful clinical management [17,18]. Despite decades of research, there remains a lack of consensus on the optimal treatment of this disorder [19]. Some guidelines recommend psychotherapeutic and drug-based interventions; however, remission and relapses persist, often leading to chronicity [20,21]. This is particularly concerning, as an estimated 50% of PTSD cases followed up in long-term studies evolve to chronicity [22,23]. The concept that “time heals wounds” only holds true for a limited number of individuals with PTSD, which strengthens the notion that important biological abnormalities predate the traumatic event and predispose to disorder severity [24].

The occurrence of trauma leading to PTSD and elevated stress may cause deviations in homeostasis, leading to pathogenic biological alterations. Persistent stress triggers an adaptive response termed “allostasis,” in which the organism attempts to cope and restore homeostatic balance through hormonal and physical dynamic changes [25]. However, allostasis is insufficient to explain the consequences of stress in an organism. The term “allostatic load” was thus coined to define the costs of chronic stress exposure associated with elevated or oscillating biological responses [26]. The “costs” of allostatic load are reflected in numerous alterations in endocrinal and immune reactivity, neural circuitry dysregulation, and molecular and physiological deterioration described in the PTSD literature [27].

As stress and challenges involved in PTSD may accumulate allostatic load in the organism and cause dysregulation of biological systems, it is crucial to investigate parameters of neuroprogression in individuals affected by the disorder. Neuroprogression, a term initially developed to describe impairment and neuroanatomical alterations in patients with bipolar disorder, is defined as a pathological brain reorganization that occurs concomitant with the decline in clinical health over the course of the disease, typically reflecting patterns consistent with accelerated biological aging [28]. As previously mentioned, substantial evidence supports the association of PTSD with adverse health outcomes. Only recently has PTSD begun to be more closely linked to biological markers of accelerated aging via measurement of telomere shortening [29] and DNA methylation [30], and in analyses of inflammation [31] and immune responses [32].

### Objectives

We designed a thematic research project to evaluate whether the traumatic events and onset of PTSD are associated with accelerated aging in individuals in the early stages of PTSD who are not chronically ill (medically and psychiatrically). We aim to enroll women in the study to better understand the effects of PTSD in this sex, given the high prevalence and severity of PTSD following sexual violence against women.

At baseline we will compare data from the PTSD group with a healthy non-PTSD control group of women with no sexual trauma history. We hypothesize that the toxicity of the increased allostatic load caused by PTSD symptoms leads to neuroprogression, even if the onset of PTSD is recent (less than 6 months). We hypothesize that we will observe:

- Poor treatment outcomes associated with neuroprogression measures.
- Reduced corpus callosum, hippocampus, and anterior cingulate cortex volume associated with increased allostatic load, suggesting that neuroprogression occurs in the early stages of PTSD.
- Alterations in telomere length and in methylation profile of long interspersed nuclear element-1 (LINE-1) regions.
- Downregulation of gene expression.
- Worse sleep quality, reduced rapid eye movement (REM) sleep, and alterations in the heart rate variability during sleep.
- Impairment of executive functions, memory, and attention associated with PTSD severity and negative outcomes.
- Hypothalamic–pituitary–adrenal imbalance, with unregulated cortisol and adrenocorticotrophic hormone levels.
- Persistent inflammatory response with increased levels of inflammatory markers.

Further, we aim to implement a randomized clinical trial (RCT) to compare 2 therapeutic interventions: interpersonal psychotherapy adapted for PTSD versus sertraline. In comparing the 2 interventions, we aim to verify whether treatment response is associated with neuroprogression measures.

## Methods

### Overall Study Design

**Figure 1:**
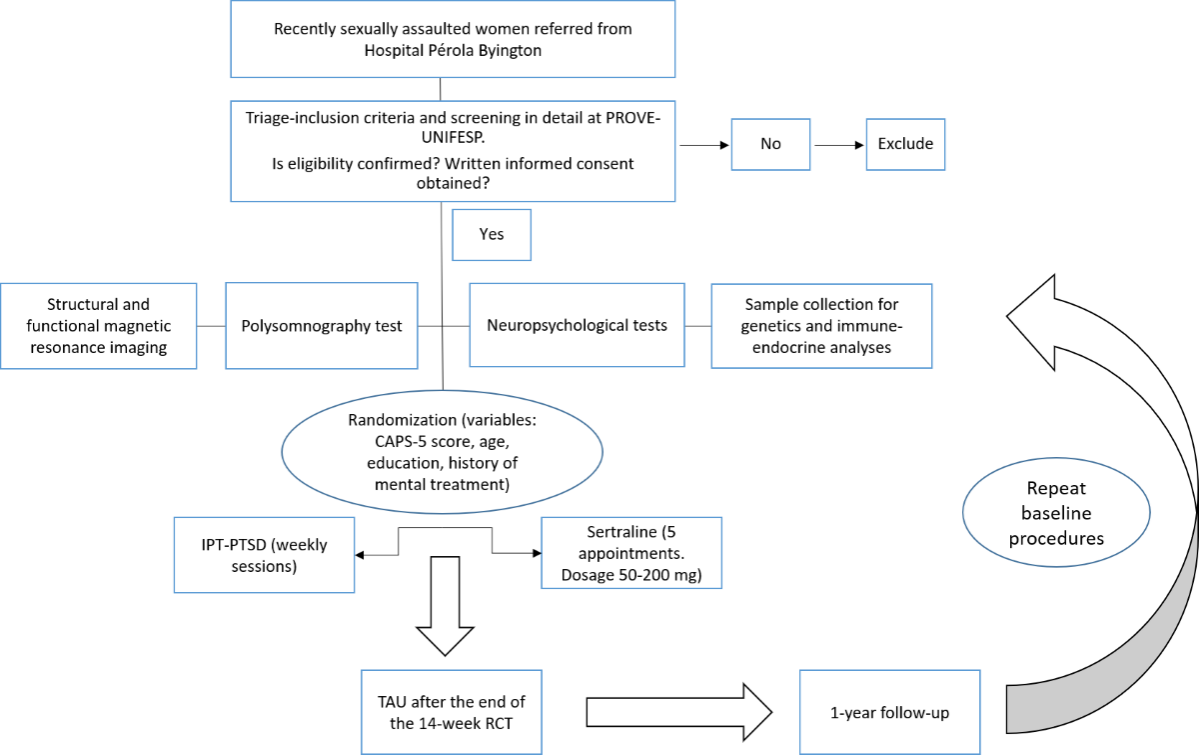
Research process flowchart. IPT: interpersonal psychotherapy; PROVE-UNIFESP: Service for Research and Care on Violence and PTSD; PTSD: posttraumatic stress disorder; RCT: randomized clinical trial; TAU: treatment as usual; UNIFESP: Universidade Federal de São Paulo (Federal University of São Paulo).

The thematic research project has a cross-sectional and longitudinal study design with multiple time-point assessments. Hospital Pérola Byington (HPB), the largest public health center facility in São Paulo that offers gynecological care for women following sexual assault, will refer all women eligible to participate in the research. Sexually assaulted women admitted to HPB will initially be requested by HPB staff to complete the National Stressful Events Survey Short Scale for PTSD-Short Scale (NSESSS-PTSD), a brief self-report PTSD screening scale. The Universidade Federal de São Paulo (Federal University of São Paulo) (UNIFESP) team will contact women by telephone to assess initial eligibility criteria. Medical records at HPB will be assessed. An appointment for screening with trained psychologists and psychiatrists at UNIFESP will be scheduled. If eligibility is confirmed, participants will be requested to undergo the following procedures: (1) structural and functional magnetic resonance imaging (MRI); (2) neuropsychological testing; (3) in-laboratory polysomnography; (4) saliva and blood sample collection for inflammatory and immune marker analyses; (5) blood sample collection for genetic analyses. After completing these procedures, all women will be randomized to receive intervention (either ITP-PTSD or sertraline) in a 14-week RCT. At the end of the 14-week RCT, women will undergo treatment as usual. After completing 1 year of treatment in the project, patients will be invited to repeat all baseline procedures, that is, the examinations and testing 1-5 described above (1-year follow-up; Figure 1).

The thematic research project has obtained a 5-year grant from the Fundação de Amparo à Pesquisa do Estado de São Paulo (FAPESP 2014/12559-5). The research will be conducted at the Service for Research and Care on Violence and PTSD (PROVE-UNIFESP) in the Department of Psychiatry at Escola Paulista de Medicina, UNIFESP. It will rely on the cooperation of different UNIFESP departments: the RCT and screening will occur at PROVE-UNIFESP; MRI will be conducted in the Department of Diagnosis in Neuroimaging; saliva and blood sample collection for genetic and inflammatory investigations, as well as the neuropsychological testing, will occur at the Associação Fundo de Incentivo à Pesquisa (AFIP-UNIFESP). The Department of Morphology and Genetics will perform DNA extraction and all genetic analyses. The Instituto do Sono-AFIP will perform in-laboratory polysomnography testing.

### Participants

#### Patient Recruitment

We estimate inclusion of 100 patients and 100 matched healthy controls who fulfill inclusion criteria. All participants must sign an informed consent form approved by the UNIFESP Committee Board, in compliance with the Code of Ethics of the World Medical Association (Declaration of Helsinki). Participation in the study will be voluntary without financial compensation. Participants will be compensated only for their public transportation expenses. If at any point in this research, any participant experiences severe symptomatic worsening, or no longer wishes to participate in the study, we will offer immediate standard treatment in our outpatient service.

#### Inclusion Criteria

Eligible women are those 18-45 years old with a definite diagnosis for PTSD according to DSM-5, who experienced nonpartner sexual violence between 1 and 6 months prior to study inclusion and developed PTSD following this particular traumatic event. We conceptualized sexual assault as rape (through force or threat), attempted rape, and drug-facilitated rape. Comorbidity with major depression, anxiety disorders, and borderline personality disorder will not be considered exclusion criteria.

#### Exclusion Criteria

Women excluded from study participation are those outside the study age range. Further exclusion factors are infection with HIV or other sexually transmissible diseases, acute clinical illness, unstable medical condition, neurological disorders, schizophrenia, bipolar disorder, current use of corticosteroid medication, menopausal symptoms, and substance abuse or dependence not in remission for the last 6 months. We will carefully select patients who are not undergoing any psychological/psychiatric treatment or taking psychotropic medication. Pregnant women will be excluded from the study.

#### Selection of Healthy Controls

We intend to enroll 100 female age-matched healthy controls selected from the community with no PTSD and no history of sexual assault who voluntarily comply with study participation. Educational level and socioeconomic characteristics will be taken into consideration to match the PTSD group. All controls will be assessed for eligibility at PROVE-UNIFESP and will undergo the same baseline procedures as the PTSD group: that is, psychometric assessments, MRI, neuropsychological testing, polysomnography, evaluation of immune and endocrinal parameters, and genetic analyses. We will exclude controls diagnosed with psychiatric disorders, currently using psychotropic medication(s), or undergoing psychotherapy. Controls will not undergo treatment and will not be followed up. Controls must not have familial relation to enrolled study patients. The same exclusion criteria for patients will be applied to controls.

### Measures

#### Full Sociodemographic Inventory and History of the Sexual Assault Episode

We developed a detailed sociodemographic inventory to collect relevant sociodemographic characteristics of our participants. We aim to collect the following data on the sexual violence episode: (1) type of violence (rape, attempted rape, or drug-facilitated rape); (2) whether the perpetrator(s) was (were) known (family member, friend, or acquaintance) or unknown; (3) location of the assault (eg, home, public place, working place), precise date, and time of the assault; (4) whether the victim chose to notify the police authorities, and if so, whether an investigation is ongoing; and (5) detailed description of the assault. We will also monitor whether participants adhere to gynecological treatment offered at HPB.

#### Clinician-Administered PTSD Scale for DSM-5 (CAPS-5)

This assessment is the gold standard to assess PTSD diagnostic status and symptom severity. The clinician administers this instrument as a structured interview, comprising 30 items assessing both the frequency and the intensity of PTSD symptoms and trauma-associated variables using a frequency/severity scale varying from 0 (never/not severe at all) to 4 (most of the time/severe). Based on a large-scale psychometric study, its authors state that the Clinician-Administered PTSD Scale for DSM-5 (CAPS-5) presented substantial evidence of both its validity and reliability as a measure for PTSD symptoms; it has been adapted to Brazilian Portuguese [33,34].

#### Mini International Neuropsychiatric Interview (MINI)

This structured diagnostic interview is designed for clinical practice and research in psychiatric and primary care settings. The Mini International Neuropsychiatric Interview (MINI) provides accurate psychiatric diagnoses (psychosis, mood, anxiety, personality, and PTSD) and is widely used in international psychiatry [35].

#### Beck Depression Inventory (BDI)

This self-report instrument is a 21-item questionnaire measuring clinical depression. Each of the 21 multiple-choice questions presents 4 alternatives varying in level of depressive severity (minimal, mild, moderate, and severe). The score consists of the sum of the most severe alternative chosen for each question [36,37].

#### Beck Anxiety Inventory (BAI)

This 21-item self-report inventory assesses anxiety symptoms. Patients must evaluate how much each anxiety symptom applies to their condition on a severity scale from 0 to 3. The score sums the individual items, classifying the severity of anxiety as minimum, mild, moderate, or severe [38].

#### Childhood Trauma Questionnaire (CTQ)

This self-report instrument for adults and adolescents investigates 5 early stage abuse and negligence experiences. It investigates physical, emotional, and sexual abuse, as well as physical and emotional neglect in childhood. The respondent indicates, on a 5-point Likert scale, the frequency of 28 different childhood situations [39].

#### Peritraumatic Dissociative Experiences Questionnaire (PDEQ)

This is a 10-item measure of dissociative symptoms experienced during or immediately after a traumatic event. The self-rated Peritraumatic Dissociative Experiences Questionnaire (PDEQ) uses a 5-point Likert scale from 1 (not at all true) to 5 (extremely true) [40].

#### The Clinical Global Impression—Severity Scale (CGI-S) and Clinical Global Impression—Improvement Scale (CGI-I)

These 7-point scales reflect the clinician’s evaluation. The GCI-S requires the clinician to rate the severity of the patient’s mental illness based on the clinician’s experience with patients with the same diagnosis. Patients are assessed for disease severity rated from 1 (normal) to 7 (extremely ill). The GCI-I requires the clinician to assess how much the patient’s condition has improved or worsened relative to the baseline of the intervention. It is rated from 1 (very much improved) to 7 (very much worse) [41].

#### PTSD Checklist for DSM-5 (PCL-5)

This 5-point Likert scale is a 20-item self-report assessing the 20 DSM-5 PTSD symptoms. Responses vary according to how much each symptom of PTSD has bothered the individual in the last 4 weeks, rated from 0 (not at all) to 4 (extremely) [42].

#### Life Event Checklist for DSM-5 (LEC-5)

This self-report measure assesses the occurrence of traumatic events in an individual’s lifetime. The Life Event Checklist for DSM-5 (LEC-5) assesses exposure to 16 events that may potentially lead to PTSD. An additional item encompasses a further extremely stressful event not captured by the other 16 items. The LEC-5 yields no total score and lacks a recognized interpretation protocol, but the instrument is commonly used in combination with other measures, such as the CAPS-5 or PTSD Checklist for DSM-5 (PCL-5), to establish actual occurrence of DSM-5 criterion A traumatic events [43].

#### Tonic Immobility Scale (TIS)

This self-report instrument was originally designed specifically to evaluate the presence and severity of tonic immobility in sexually assaulted women. The Tonic Immobility Scale (TIS) reflects physiological and behavioral features that accompany tonic immobility during the traumatic event and comprises 2 parts. The first part assesses multiple dimensional tonic immobility responses; the second assesses victim behaviors in contextual assault circumstances [44].

#### Structured Clinical Interview for DSM-5 for the Diagnosis of Borderline Personality Disorder (SCID-5)

This is a semistructured interview administered by the clinician or a trained mental health professional to assess the major DSM-5 diagnoses and symptom severity dimensions of both current and lifetime occurrence of mental disorders [45]. Specific segments for borderline personality disorder, common comorbidity with PTSD, and influential aspects of PTSD treatment outcomes will be used.

#### World Health Organization’s Quality of Life Assessment (WHOQOL-BREF)

This abbreviated self-rated questionnaire, designed with international cooperation for global cross-cultural use, assesses quality of life. Its 25 facets are scored in environmental, social, physical, and psychological domains [46].

#### The Revised Adult Attachment Scale (RAAS)

This 5-point Likert scale measures adult attachment and assesses close interpersonal relationships. The 18 items range from 1 (not at all characteristic) to 5 (very characteristic of me) and are classified into 3 subscales: closeness, dependency, and anxiety [47].

#### The Sheehan Disability Scale (SDS)

This brief 3-item self-report measures the impairment and disruption caused by symptoms to work, family, and social functioning. Patients self-rate on a scale from 0 (not at all) to 10 (extremely) [48].

#### Modified Fatigue Impact Scale (MFIS)

This 21-item scale assesses the perceived effects of fatigue on quality of life in physical, cognitive, and psychosocial domains. It is mainly used in patients with chronic diseases. The Modified Fatigue Impact Scale (MFIS) uses a 5-point Likert scoring system from 0 (never) to 4 (almost always) [49].

#### Pittsburgh Sleep Quality Index (PSQI)

This self-administered, reliable tool evaluates sleep quality and possible disturbances in the previous month. It is used in both clinical practice and research. The 19 Pittsburgh Sleep Quality Index (PSQI) individual items generate 7 component scores: subjective sleep quality, sleep latency, sleep duration, habitual sleep efficiency, sleep disturbances, use of sleeping medication, and daytime dysfunction [50]. We will use an addendum of the PSQI designed explicitly to measure PTSD-related sleep dysfunction [51].

#### Epworth Sleepiness Scale (ESS)

This self-report questionnaire measures the occurrence and intensity of daytime sleepiness. The Epworth Sleepiness Scale (ESS) evaluates the probability of falling asleep in 8 situations involving daily activities. Total score ranges from 0 to 24; scores over 10 suggest excessive daytime sleepiness [52].

#### Insomnia Severity Index (ISI)

This brief self-report instrument measures patient perception of his or her insomnia. The Insomnia Severity Index (ISI) targets the subjective symptoms and consequences of insomnia. The ISI also measures the degree of concerns or distress caused by those difficulties. The ISI comprises 7 items assessing the severity of sleep-onset and sleep maintenance difficulties, satisfaction with current sleep patterns, interference with daily functioning, noticeability of impairments attributed to sleep problems, and degree of distress or concern caused by the sleep problems. Each item is rated on a scale from 0 to 4, and the total score ranges from 0 to 28 [53].

#### Alcohol Use Disorders Identification Test (AUDIT)

This 10-item questionnaire was developed by the World Health Organization to identify patients with recent heavy drinking or alcohol dependence. The Alcohol Use Disorders Identification Test (AUDIT) score ranges from 0 to 40. An AUDIT score of 8 indicates a pattern of harmful alcohol consumption; 16 to 19 indicates alcohol abuse, and 20 shows probable dependence [54].

#### Alcohol, Smoking, and Substance Involvement Screening Test (ASSIST)

This interview developed by the World Health Organization identifies history and current (past 3 months) use of substances. The Alcohol, Smoking, and Substance Involvement Screening Test (ASSIST) assesses cannabis, cocaine, amphetamine-type stimulants, inhalants, sedatives, hallucinogens, opioids, other miscellaneous drugs, and alcohol or tobacco use. After responding regarding the lifetime use of all substances investigated, participants respond regarding recent use on a 5-point scale (frequency) ranging from “never” to “daily or almost daily” [55].

### Detailed Procedures

#### Magnetic Resonance Imaging

Brain MRI scans will be performed using a 3T Philips Achieva scanner with a 32-channel head coil. The MRI protocol consists of a localizer; coronal T2; volumetric T1 (repetition time [TR] = 2000 ms; echo time [TE] = 3 ms; inversion time = 1100 ms; field of view = 240 × 240 × 180 mm^3^; matrix size = 240 × 240 × 180); axial fluid-attenuated inversion recovery; diffusion tensor imaging (weighted images acquired using a single-shot, spin-echo, echo‐planar imaging with b=1000 s/mm^2^ with 31 uniformly distributed, noncolinear direction images, plus 1 additional image acquired with nondiffusion weighting [b=0 s/mm^2^]); 2D multislice gradient echo sequence with magnitude and phase images (TR = 500 ms, TE = 2.0 and 4.3 ms; flip angle = 30°, fat saturation = off, water fat shift = 0.3 pixels, bandwidth (BW) = 1446.8 Hz/pixel); resting-state functional MRI (isotropic resolution 3 × 3 × 3 mm^3^, TR = 2 s, TE = 30 ms, 300 volumes); and 3 MRI spectroscopy sequences: (1) in the anterior cingulate gyrus using chemical shift imaging (multivoxel spectroscopy) with a 2D PRESS sequence (TE/TR = 45/2000 ms, 1024 samples, BW = 2 kHz, voxel size = 10 ×10 × 15 mm, matrix = 8 × 8 voxels, oversampled to 13 × 12 to avoid aliasing, average = 1, total scan time = 3:30 min); (2) multiecho single-voxel PRESS (TR = 2000, TE = 30, 40, 50, 60, ..., 250, 260 ms [24 TEs in total], 2048 samples, BW = 2 kHz, voxel size = 20 × 20 × 20 mm, average = 8, total scan time = 8:48 min) according to a protocol by Hurd and colleagues [56]; and (3) conventional long TE single-voxel PRESS (TE/TR = 144/2000 ms, 2048 samples, BW = 2 kHz, voxel size = 20 × 20 × 20 mm with the same positioning as that in [2], average = 96, total scan time = 3:48 min). All MRI protocols include the acquisition of an unsuppressed water reference spectrum. Details on imaging preprocessing and analyses will be provided in specific studies from this cohort.

#### Neuropsychological Testing

Application of a neuropsychological battery will be composed of the following subtests: Wechsler Abbreviated Scale of Intelligence (WASI) Vocabulary and Matrix Reasoning, Digit Subtests of the Wechsler Adult Intelligence Scale (WAIS-III), Rey Auditory Verbal Learning Test (RAVLT), Spatial Span Subtest of the Wechsler Memory Scale (WMS), III Edition, abbreviated version of the Wisconsin Test, Concentrated Attention Test (D2), Prospective Memory Subtest of the NEUPSILIN Scale, Five-Digit Test, and the Stroop Test (Trenerry’s version).

#### Polysomnography

Participants will undergo 1 night of polysomnography recording in the Instituto do Sono-AFIP at UNIFESP. Recordings will be conducted using Embla N7000 (Embla Systems, Inc.). In the polysomnography test, we will measure brain activity using electroencephalogram electrodes, monitor eye movements using electrooculography electrodes, measure muscle tone using electromyography electrodes, measure heartbeat using electrocardiograph electrodes, and monitor rate of respiration using respiratory monitors. The following sleep variables will be determined: REM sleep latency; sleep onset latency; total sleep time; wake after sleep onset; sleep efficiency; percentages of total sleep composed of N1, N2, N3, and REM sleep; arousal index; number of arousals per hour; periodic limb movements in sleep indices with and without arousal; number of limb movements per hour with and without arousal; apnea–hypopnea index; number of apneas and hypopneas per hour; and REM sleep density. A power spectral analysis of heart rate variability will be performed from the electrocardiogram signal collected throughout sleep and will assess the quantitative contribution of high frequency (0.15-0.4 Hz), low frequency (0.04-0.15 Hz), and very low frequency (0.003-0.04 Hz) components to the total variance.

#### Immune and Endocrinal Parameters

Results for interleukin-6, interleukin 1-β, monocyte chemoattractant protein-1, tumor necrosis factor-α, and C-reactive protein will be obtained from blood samples of the participants at the AFIP-UNIFESP at 7:00 am after the polysomnographic examination. We will use ethylenediaminetetraacetic acid (EDTA) tubes, and the plasma will be stored at –20°C.

Salivary cortisol samples will be collected using the Salivette kit with a synthetic swab. All participants will be provided instructions according to the kit protocol. Saliva will be collected at home 4 times: at 10 p.m. before sleeping and the next day at 6:30 a.m., 7 a.m., and 7:30 a.m.

A second collection of salivary cortisol will be performed at the Instituto do Sono-AFIP at UNIFESP. Professionals will collect saliva at 10 p.m. (prepolysomnographic examination), 6:30 a.m., 7 a.m., and 7:30 a.m. (postpolysomnographic examination).

For C-reactive protein, adrenocorticotrophic hormone, vasopressin, aldosterone, and cortisol analyses, we will use a high-sensitive enzyme-linked immunosorbent assay kit (ELISA). The MILLIPLEX MAP panel based on the Luminex xMAP technology will be used to obtain cytokine results.

#### Genetics

Peripheral blood of the participants will be collected in 2 EDTA tubes for DNA isolation and 2 PAXgene RNA tubes for RNA analysis. DNA and RNA extraction will be performed using Gentra Puregene (Qiagen) and PAXgene blood RNA isolation kits, respectively, according to the manufacturer’s instructions.

The DNA samples will be used to perform different genetic/epigenetic analyses such as genotyping, measurement of telomere length, and methylation profile over LINE-1 regions. The genotyping of individuals will be performed using the Infinium Global Screening Array BeadChip (Illumina) with approximately 640,000 markers, including rare, common variants, and exonic, intronic, nonsense, missense, indel markers. Telomere length will be measured by quantitative polymerase chain reaction, according to a protocol previously described by Cawthon [57], which consists of determining the relative ratio between the telomere region copy number (T) and a single-copy gene (S – albumin gene) using a relative standard curve. The methylation profile of LINE-1 transposable elements was verified by quantification of global methylation of these fragments using polymerase chain reaction followed by sequencing using Pyrosequencing technology.

The RNA samples will be used to investigate the blood transcriptome by sequencing using the NextSeq 500/550 High Output Kit (version 2.5; Illumina).

### Randomized Clinical Trial: Interpersonal Psychotherapy Adapted for PTSD Versus Sertraline

We designed a 14-week RCT comparing IPT adapted for PTSD to sertraline. Our plan is to evaluate the relative efficacy of both interventions, comparing treatment outcome, patient adherence, and tolerability for women in the early stages of PTSD following sexual trauma. Sertraline is a widely used, usually well-tolerated selective serotonin reuptake inhibitor (SSRI) effective as an antidepressant and anxiolytic, and one of the 2 SSRIs FDA approved to treat PTSD [58,59]. However, several guidelines consider antidepressants to be of secondary importance to psychotherapy for the treatment of PTSD [60], and many SSRI-treated patients do not achieve remission [61].

Interpersonal psychotherapy adapted for PTSD is a non-exposure, nontrauma-focused brief therapy designed to minimize the high attrition rates observed in patients undergoing exposure therapies. Many traumatized individuals refuse or are unable to tolerate treatments such as prolonged exposure, as they require facing reminders of the traumatic event(s), which often trigger fear, avoidance, and intolerable stress [62]. The aversive aspects of exposing patients to their traumatic memories and cues may elevate attrition, reducing treatment efficacy. Interpersonal psychotherapy adapted for PTSD explores interpersonal and commonly severe social aftereffects of trauma, not the trauma itself [63]. In one RCT, interpersonal psychotherapy adapted for PTSD produced superior results to prolonged exposure in treatment retention and symptom improvement in chronic sexual assault survivors [64].

Interpersonal psychotherapy adapted for PTSD is delivered in 3 phases: (1) assessment of PTSD diagnosis and patients’ interpersonal context, including meaningful past and present relationships. The therapist offers a life crisis formulation that provides the therapeutic focus (2-3 sessions); (2) the therapist helps patients to resolve the focus, eliciting and validating patients’ thoughts and feelings and encouraging them to take appropriate social risks to improve abilities to assert wishes and needs in relationships (8-9 sessions); (3) the therapist terminates treatment, calling attention to patients’ gains during the process, and reinforces positive roles (eg, as a survivor rather than a victim). Positive and painful aspects of the intervention are addressed to help patients finalize the psychotherapy process (3 sessions) [63,65]. Part of adapting IPT for PTSD involves helping numbed patients attune to their emotions, and to recognize them not as dangerous but as helpful interpersonal signals.

In the pre-enrollment consent process, patients will be told about the study design, and that they may not choose between the treatments. Both arms will have a 14-week duration and will be conducted entirely at PROVE-UNIFESP. Patients nonresponsive to treatment will not be crossed over to the other arm, and will be excluded from the research if they become suicidal or if their symptoms become more severe. A brief description of the methods applied in both arms is as follows:

- Interpersonal psychotherapy adapted for PTSD will comprise weekly 50-minute sessions with an experienced, well-trained therapist who will be supervised every week by 2 expert interpersonal psychotherapy adapted for PTSD therapists. The sessions will be audiotaped with patient consent, and the interpersonal psychotherapy adapted for PTSD team will study the recordings to assess therapy quality by rating therapist verbalizations rather than patient responses. Patients will be seen briefly 5 times during the 14-week treatment by a psychiatrist, who will have the option to introduce low dosage of the following sedative medications: quetiapine (25-50 mg), risperidone (1-2 mg), or zolpidem CR (12.5 mg). Interpersonal psychotherapy adapted for PTSD therapists and clinicians will maintain confidentiality.
- Sertraline will be administered using an initial dosage of 50 mg that may be increased to 200 mg over the 14-week treatment. Patients will be seen 5 times during the 14-week trial by psychiatrists experienced in treating patients with PTSD. Additional dosages of quetiapine (25-50 mg), risperidone (1-2 mg), or zolpidem CR (12.5 mg) may be used.

Different psychiatrists will evaluate the patients in either arm of the trial. The additional drugs mentioned above (quetiapine, risperidone, and zolpidem CR) will be prescribed only if patients exhibit high levels of anxiety, fear, or severe insomnia.

**Figure 2:**
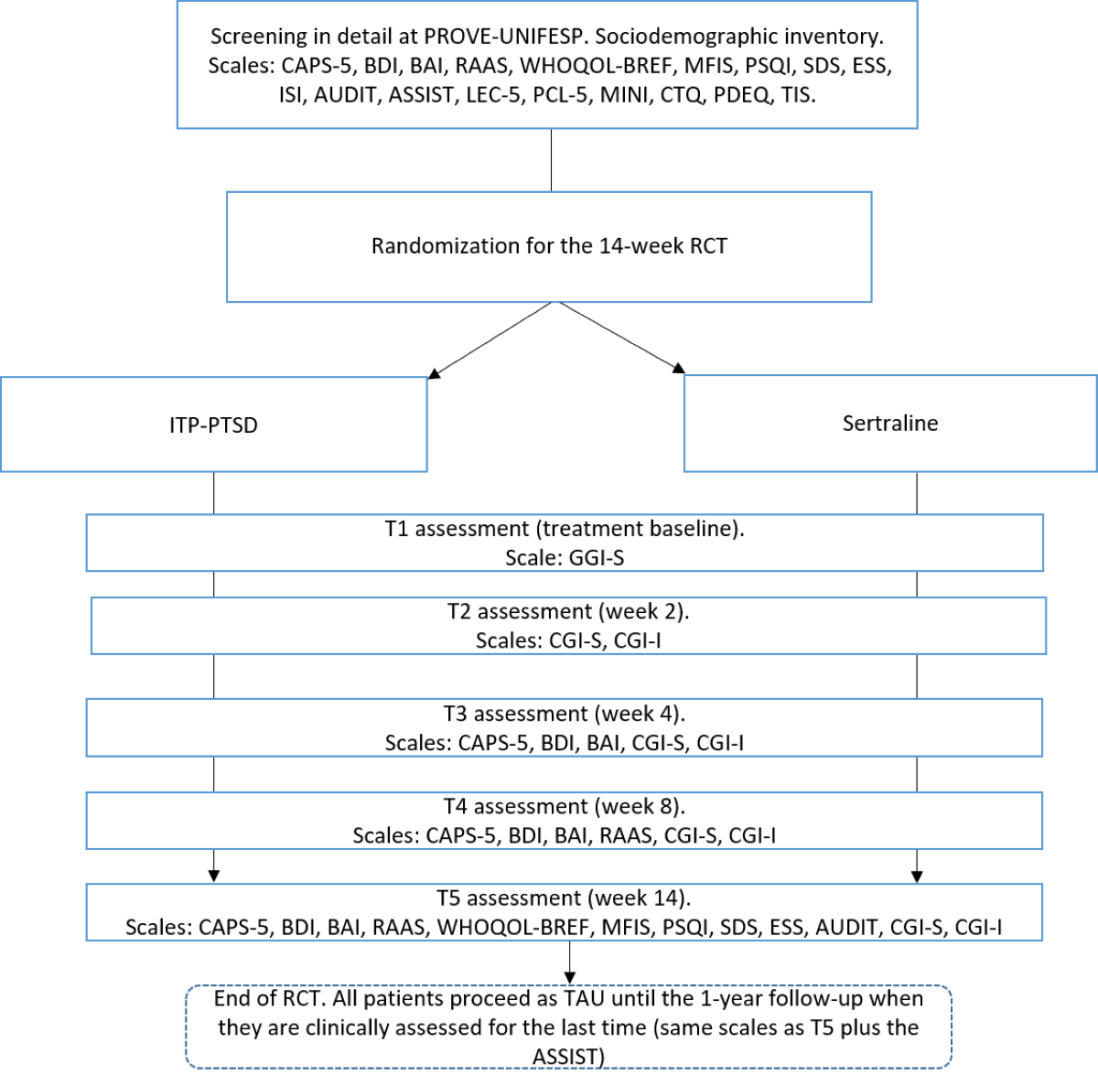
Clinical assessment flowchart. ASSIST: Alcohol, Smoking and Substance Involvement Screening Test; AUDIT: Alcohol Use Disorders Identification Test; BAI: Beck Anxiety Inventory; BDI: Beck Depression Inventory; CAPS-5: The Clinician-Administered PTSD Scale for DSM-5; CGI-I: The Clinical Global Impression—Improvement Scale; CGI-S: The Clinical Global Impression—Severity Scale; CTQ: Childhood Trauma Questionnaire; ESS: Epworth Sleepiness Scale; IPT: interpersonal psychotherapy; LEC-5: Life Event Checklist for DSM-5; MINI: Mini International Neuropsychiatric Interview; MFIS: Modified Fatigue Impact Scale; PCL-5: PTSD Checklist for DSM-5; PDEQ: Peritraumatic Dissociative Experiences Questionnaire; PROVE-UNIFESP: Service for Research and Care on Violence and PTSD; PSQI: Pittsburgh Sleep Quality Index; PTSD: posttraumatic stress disorder; RAAS: The Revised Adult Attachment Scale; RCT: randomized clinical trial; SDS: The Sheehan Disability Scale; TAU: treatment as usual; TIS: Tonic Immobility Scale; WHOQOL-BREF: World Health Organization’s Quality of Life Assessment.

The clinical assessments of the study are presented in Figure 2. Longitudinal assessments will occur at week 2 (CGI-S and CGI-I), week 4 (CAPS-5, Beck Depression Inventory [BDI], Beck Anxiety Inventory [BAI], CGI-S, and CGI-I), week 8 (CAPS-5, BDI, BAI, The Revised Adult Attachment Scale [RAAS], CGI-S, and CGI-I), and week 14 (CAPS-5, BDI, BAI, RAAS, WHOQOL-BREF, MFIS, PSQI, SDS, ESS, AUDIT, CGI-S, and CGI-I). The CGI-S and the CGI-I will be used by the clinicians in all appointments. The research team will administer the other scales in a separate meeting with patients. Assessors will be blind to treatment condition and will not provide any treatment. The same measures will be repeated at 1-year follow-up with the addition of the ASSIST.

### Sample Size Power Calculation for the RCT and Randomization

A sample size of 84 patients with PTSD (42 in each group) could be adequate considering a difference between 2 independent proportions. To obtain the sample size, we fixed a probability of a type I error of .05 (level of significance), and the power of the test of 0.8 (probability of type II error of .2) for a bicaudal test. In addition, we considered an index of 0.6 (60%) of response for active treatment for PTSD, and a placebo effect of 0.3 (30%). We increased the number by 20% in anticipating dropouts (n=102) and we will use sequential randomization to prevent an imbalance between treatment groups for known factors (symptom severity, age, educational level, history of previous treatment) that influence prognosis or treatment responsiveness. The sequential randomization also prevents type I error and improves the power of the test for small trials (<400 patients).

We will randomize eligible patients to either interpersonal psychotherapy adapted for PTSD or sertraline using a method developed to balance prognostic factors in psychiatric clinical trials [66]. This method allows allocation into 2 homogenous groups. Randomization will be performed by an independent statistician who will generate an allocation sequence and will keep the assignment schedule in a separate computer. The following variables will be used: CAPS-5 total score, age, educational level, and whether there is history of prior psychological/psychiatric treatment.

### Statistical Analyses Planned for the RCT

We will use a generalized estimating equation [67] to evaluate the effects of time and group randomization (ie, sertraline or interpersonal psychotherapy adapted for PTSD) on 4 different outcomes: CAPS-5 score, BDI, BAI, and CGI-I. Each patient in the RCT will receive 4 time-point longitudinal assessments. We will apply a first-order autoregressive effect working correlation to consider carryover effects of the PTSD symptoms across time [68]. The first-order autoregressive working correlation matrix will be used to deal with the within-subject effect, as it is expected that measurements taken further apart are less correlated than those taken closer together.

Patient attrition is commonly observed and expected in multiple time-point assessment clinical trials. To minimize the impact of patient dropout, an intention-to-treat analysis must be performed [69]. We will use 2 different techniques to deal with missing data when estimating time and group allocation, which are our main covariates of interest. The first technique will be a full-information maximum likelihood, which is the default estimator (maximum likelihood) to accommodate missingness when doing generalized estimating equation. This approach is important due to the long format of the data set and when (at least) baseline measures are available in the outcome. The second technique is a flexible approach to deal with missing data when implementing clinical trials [70]. It replaces missing data with one or more specific values, to allow statistical analysis that includes all participants and not just those who do not have any missing data. We will run all analyses using SPSS (version 24; IBM).

### Ethics Approval and Consent to Participate

The clinical trial of this study was registered at Brazilian Clinical Trials Registry (registration number RBR-3z474z; registration date: March 24, 2015).

The Institutional Review Board of the UNIFESP approved the study protocol. Written informed consent will be obtained, following statement of compliance with the Code of Ethics of the World Medical Association (Declaration of Helsinki) and the standards of the Review Board and granting agency.

## Results

Data collection started in early 2016 and will be completed by the end of the first semester of 2020. Analyses will be performed soon afterward, followed by the elaboration of several articles. Articles will be submitted in early 2021.

## Discussion

Sexual violence against women is a great concern to society and among health providers. It often leads to prolonged and severe mental health consequences such as PTSD. Most studies of the associations between PTSD and neuroprogression evaluate alterations in chronic patients, particularly older male veterans. More research is warranted to investigate the neurobiology of recent PTSD in women. With this thematic research project, we will investigate the occurrence of accelerated biological aging in our patients by assessing markers associated with aging through neuroimaging, genetics, neuropsychological testing, sleep disturbances, immune and inflammatory alterations, and how they relate to clinical outcomes and treatment response.

The main study strength is that it addresses important knowledge gaps in understanding the extent to which PTSD triggered by recent exposure to sexual assault affects neuroprogression in young women. To the best of our knowledge, this is the first thematic research to investigate accelerated aging as a consequence of sexual assault–related PTSD in women from a developing country. Sexual assault in Brazil has reached staggering numbers. Although there is a lack of studies to estimate more precisely the prevalence of sexual assault in the Brazilian population, previous evidence pointed to half a million women being sexually assaulted every year [71]. As sexual assault often leads to PTSD, and violence against women is on the rise in many countries, this is a global public health concern.

Another study strength is its investigation of the effectiveness of interpersonal psychotherapy adapted for PTSD, a non-exposure psychotherapy, in a sample of sexually assaulted Brazilian women. As successful treatment of PTSD can be challenging, we expect our contribution will deepen the discussion of treatment options through implementing an RCT contrasting sertraline with interpersonal psychotherapy adapted for PTSD. This is the first RCT to compare IPT to SSRI for PTSD. Following up our research patients will also permit to assess correlations between treatment response and neuroprogression measures.

The study has limitations. As published evidence on biological markers in the early stages of PTSD is lacking, we are unable to calculate the sample size to power the eventual biomarker alterations found in our sample. Based on the previous literature on PTSD interventional studies, we can provide a sample size calculation for the RCT, but not for all investigations that will be conducted in the thematic research project. Furthermore, investigation of aging markers in the early stages of PTSD and in a relatively youthful patient sample may prove too early to be detectable. However, it is warranted to investigate whether PTSD following sexual assault may accelerate deterioration of biological health parameters in young women. Our approach may fuel further research to investigate initial biological alterations in patients with PTSD before evolution to chronicity that could be used as biomarkers in the near future. Another limitation is that social stigma may discourage women from seeking mental treatment following sexual assault. Accordingly, refusal to participate in the study may be high and impede achieving the hoped-for quantity of patients we aim to enroll. Another concern is the possible elevated attrition rates, a common problem in the treatment of PTSD. A high attrition rate could limit the performance of the RCT, and weaken the longitudinal analyses of neuroprogression in our sample and its associations with treatment response.

## Abbreviations

ASSIST: Alcohol, Smoking and Substance Involvement Screening Test
AUDIT: Alcohol Use Disorders Identification Test
BAI: Beck Anxiety Inventory
BDI: Beck Depression Inventory
CAPS-5: The Clinician-Administered PTSD Scale for DSM-5
CGI-I: The Clinical Global Impression—Improvement Scale
CGI-S: The Clinical Global Impression—Severity Scale
CTQ: Childhood Trauma Questionnaire
DSM-5: Diagnostic and Statistical Manual of Mental Disorders, fifth edition
ESS: Epworth Sleepiness Scale
GEE: generalized estimating equation
HPB: Hospital Pérola Byington
ISI: Insomnia Severity Index
LEC-5: Life Event Checklist for DSM-5
LINE-1: long interspersed nuclear element 1
MFIS: Modified Fatigue Impact Scale
MINI: Mini International Neuropsychiatric Interview
MRI: magnetic resonance imaging
NSESSS-PTSD: National Stressful Events Survey Short Scale
PCL-5: PTSD Checklist for DSM-5
PDEQ: Peritraumatic Dissociative Experiences Questionnaire
PROVE-UNIFESP: Service for Research and Care on Violence and PTSD
PSQI: Pittsburgh Sleep Quality Index
PTSD: posttraumatic stress disorder
RAAS: The Revised Adult Attachment Scale
RCT: randomized clinical trial
REM: rapid eye movement
SDS: The Sheehan Disability Scale
SSRI: selective serotonin reuptake inhibitor
TIS: Tonic Immobility Scale
UNIFESP: Universidade Federal de São Paulo (Federal University of São Paulo)
WHOQOL-BREF: World Health Organization’s Quality of Life Assessment
